# Nutritional status of 6–59 months of age children is not significantly varied between households with and without home gardening practices in Zege, North West Ethiopia, 2020: community based comparative study

**DOI:** 10.1186/s12887-022-03283-5

**Published:** 2022-04-23

**Authors:** Mulat Tirfie Bayih, Zerfalem Arega, Achenef Motbainor

**Affiliations:** 1 Department of Public Health Nutrition, School of Public Health, College of Medicine and Health Sciences, Bahir Dar University, Bahir Dar, Ethiopia; 2 Department of Environmental Health, School of Public Health, College of Medicine and Health Sciences, Bahir Dar University, Bahir Dar, Ethiopia

**Keywords:** Nutritional status, Under-five children, Home gardening households, Ethiopia

## Abstract

**Background:**

Malnutrition is a critical public health issue that has been related to a significant increase in mortality and morbidity rates. Despite the fact that children are expected to benefit from home gardening products, their nutritional condition in Ethiopia, particularly in the planned study region, is not thoroughly monitored. Therefore the purpose of this is to determine the nutritional status of 6–59 months of age children between households with and without home gardening practices at Zege.

**Methods:**

A community based comparative cross-sectional study was conducted among paired mothers with 6–59 month children from February to March 2020. Data were collected using questionnaire and anthropometric measurement tools. Binary logistic regression models were used. A-*p*-value < 0.05 was used as cutoff point to declare statistically significant variables with the outcome variable.

**Result:**

Stunting and wasting among children aged 6–59 months was high and did not show significant variation between households practicing home gardening (stunting 46.1%, at 95%, CI: 40.6–51.3 and wasting 9.1%, at 95% CI: 6.2–12.7) and not practiced home gardening (stunting 50.3%, at 95% CI: 44.5–55.8 and wasting 10.1%, 95% CI: 6.8–13.8). Having low dietary diversity (AOR = 2.7; 95% CI: 1.9–3.9), Being male (AOR = 2.1; 95% CI: 1.4–3), feeding frequency < 3/day (AOR = 1.7; 95% CI: 1.1–2.4), and presence of diarrhea (AOR = 2.6; 95% CI: 1.4–4.6) were predictors of stunting. Unprotected-drinking water (AOR = 2.1; 95% CI: 1.0–4.2), not fully-immunized (AOR = 2.6; 95% CI: 1.3–5.1) and being female (AOR = 2.4; 95% CI: 1.3–4.6) were predictors for child wasting.

**Conclusion:**

stunting and wasting are highly prevalent in both home gardening and non- home gardening households’ children of the community. Promoting diversified diet, protected water source, vaccinating children, access to a healthy environment and integrated with the access of nutrition education programs are vital interventions to improve nutrition.

## Introduction

Malnutrition, defined as being either undernourished or over nourished, is a serious public-health issue that has been linked to a significant increase in the risk of mortality and morbidity [1]. Child malnutrition causes a delay in physical growth and motor development, lower intellectual quotient (IQ), greater behavioral problems, a lack of skills, susceptibility to contracting diseases, chronic illnesses in adulthood which can have an intergenerational effects, [2, 3]. Wasting, stunting, and underweight are defined as Z-scores less than − 2 standard deviations of weight for height, height for age, and weight for age, respectively [4].

Globally, 150.8 million under the age of five children were stunted, and 50.5 million children were wasted [5]. Malnutrition is an underlying cause for nearly half (45%) of child deaths, particularly in low socioeconomic communities, with more than 7 million women suffering from complications due to vitamin A deficiency and causing deaths in 6–8 percent of the children under the age of five in Africa and Asia [6]. Furthermore, the, overall under-5 mortality rate due to malnutrition was 55 deaths per 1,000 live births [7]. Under nutrition remains a major public health concern in developing countries, as a result of a combination of poor dietary consumption, infection such as diarrhea, household food insecurity, and poor sanitation practices [8]. Malnutrition is one of the most serious public health issues in Ethiopia, and one of the world's largest [9]. Stunting is extremely common in Ethiopia, ranging from (49% in Tigre to 14% in Addis Ababa), and wasting has varying figures in different parts of the country, reaching up to 21 percent in Afar [10, 11].

Despite the achievements in agricultural productivity, access to basic health services and education around the worldwide, the progress of malnutrition especially under nutrition reduction has been characterized by poor nutritional outcomes and lag in measures of social and economic progress [12]. Similarly the 2018 Global Nutrition Report finds again that the problem of malnutrition remains severe, the world is falling short of its goals, and that malnutrition in all its forms remains unacceptably high across all regions of the world [13].

Multiple strategies are required to address the issue of malnutrition, food production and food security, and home gardening practice is one of nutrition sensitive intervention systems in many developing countries and is widely used as a remedy to alleviate hunger and malnutrition [14]. Evidences suggests that home garden food production played a role in improving household dietary intake and income earning, lowering the proportion of under-five malnutrition, and increasing women’s involvement in household decision making [15–18]. Furthermore, despite the Ethiopian government's development of a Food and Nutrition Policy (FNP), a National Nutrition Program (NNP), and the Sequta Declaration to end all forms of malnutrition in children under the age of five, malnutrition remains a public health problem [19–21].

Inadequate food intake, food insecurity, inadequate child care, illness such as diarrhea and other infections, lack of sanitation, low maternal education, low socioeconomic status, low Dietary Diversity Score (DDS), and rural residency are all risk factors for child malnutrition [7, 22–24].

Despite the fact that there is information on the effects of home gardening on child nutrition, well-organized and documented evidences are lacking in Ethiopia. One reason for this evidence scarcity could be the country's limited use of home gardening practices, particularly in the proposed study area.

This study is aimed to compare the nutritional status and associated factors among children aged 6–59 months between HHs with and without home gardening practice in rural town of Bahir Dar city administration Zege North West Ethiopia.

## Methods and materials

### Study setting and Period

The study was conducted in Zege, from February 10^th^ to March 10^th^ /2020. Zege is one of the Bahr-Dar city administrations rural satellite town found 32 km far from Bahir Dar, the capital of Amara National Regional state. Zege peninsula is found around religious area and households didn’t have enough farm lands for production of diversified croups [25].

Based on 2019 population projection given from Bahir Dar city administration, the current population size of Zege is 10,083 (Male 4041, Female 6042). For administrative purpose Zege has 03 Keble’s and 2142 total HHS, 709 with home gardening and1433 without home gardening. Zege has 01 health centers and 03 health posts and each of health facility giving health care service to the population of Zege and nearby population [26].

### Study design and populations

A Community based comparative cross-sectional study was used to determine nutritional status of children aged 6–59 months and its associated factors between Households with and without home gardening at Zege rural town of Bahr-Dar city administrations northwest Ethiopia. All care givers and 6–59 month of age children with and without home gardening were included in the study.

### Sample size determination

The required sample size of the study was determined by using double population proportion formula by considering the following assumption.95% confidence level,80% power, of the study, P1 and P2 the prevalence of stunting in home gardening and non-home gardening populations, respectively.

$$\mathrm{n}=\frac{[(\mathrm{Z}1 +\mathrm{ Z}2)2*2\mathrm{pq}]}{(\mathrm{P}1-\mathrm{P}2)2}$$

p1 = prevalence of stunting of under five children with home garden 41% from previous study (South Ethiopia) [27] P2 = prevalence of stunting of under five children without home garden 52.5% from previous study (East Gojam) [8]. Finally, by taking 10% non-response rate the total sample size was **647.**

### Sampling technique and sampling procedure

First, Kebeles (the smallest administrative unit in the country) in the district were stratified based on home gardening status (home garden user and non-home garden users and non-irrigation users). The total number of 6–59 months old children in each kebeles were taken from the respective households using the registration book at health posts. Then, the calculated sample size (648), 324 for each group were selected using simple random sampling (computer generating method) technique whenever there were two or more under- five children, in the house, the youngest child was selected in order to avoid recall bias.

### Data collection tools and procedures

Different types of tools and measurements were implemented to collect the required data. Structured interviewer administered questionnaire was developed by reviewing different literature. The questionnaire has sections like socio-demographic, and/or socioeconomic characteristics, nutrition related, wash related, health related factors and anthropometric measurements. After HHs who have 6–59 months of age children were selected from health posts, then data collectors were go to house to house for interview. Four clinical nurses and two health officer were assigned for data collection and supervisor respectively.

Weight and height of children were taken using the standard anthropometric measurement procedures outlined in the measurement guide prepared by the Food and Nutrition Technical Assistance (FANTA) project in 2007 [28]. Body weight was measured using portable weighing scale in light clothing with no jackets or coats, shoes, and additional clothing to the nearest 0.1 kg. For child less than two or unable to stand was the difference between weight of the mother caring the child and the weight of the mother alone on a new calibrated portable scale. Weighing scale was calibrated before and after any measurement. Height of children was measured using a portable stadiometer with no shoes; the shoulders, buttocks, and the heels touched the vertical stand with the head in Frankfurt plane to the nearest 0.1 cm. Children with 6–23 months of age, recumbent length the nearest to 1 mm and for children 24- 59 months of age, standing height to the nearest 0.1 cm was measured.

Both height and weight was recorded with two decimal places. Age of each child was also collected from the mother and counter-checked using vaccination cards or other forms of informal recording. All anthropometric measurements were taken twice, and the average of the two measurements were calculated and recorded.

#### Dietary diversity of children

A 24-h recall method (from sun rise to sun rise) was used to assess dietary diversity practices. It was based on the mother’s recall of foods given to her child in the previous24 hours prior to the interview date. Then, minimum dietary diversity was estimated using information collected from the24-hour dietary recall. Minimum dietary diversity was fulfilled if a child had received four or more food groups from the seven WHO food groups in the last 24 h preceding the survey. Seven food groups included were grains, roots, and tubers; legumes and nuts; dairy products (milk, yogurt, and cheese); flesh foods (meat, fish, poultry, and liver/organ meats); eggs; vitamin rich fruits and vegetables; and other fruits and vegetables [29].

#### Food security status of the households

determined based on nine standard Household Food Insecurity Access Scale (HFIAS) questions that were developed for this purpose by Food and Nutrition Technical Assistance (FANTA) [30].

#### Wealth index of the households

Was determined using the Principal Component Analysis (PCA) by considering latrine, water source, household assets, live-stock, and agricultural land ownership. Quintiles of the wealth score was created to categorize households as poor, medium and rich [31].

### Data quality control

To maintain the quality of data, first, standardized data collection tools were developed in English and translated to Amharic (local language) for data collection then back to English for consistency. Pretest was done on 5% of the total sample size in other sites in order to evaluate the developed questioner. Weighing scale was calibrated before each measurement using known weight and all anthropometric measurements were taken twice, and the average of the two measurements were calculated and recorded. Two-day training was given for data collectors and supervisor prior to the actual data collection time on the selection procedure of study participants, purpose of the study, on the steps how they can give the necessary information for the participants when they start data collection. The supervisor and principal investigator were supervised and checked the completeness and quality of data daily. During data collection, questionnaires were reviewed and checked for completeness by the supervisor and principal investigators and the necessary feedback was offered to the data collectors in the next morning.

### Data processing and analysis

The collected data was coded, entered and cleaned using Epi data version 3.02 and exported to SPSS version 23 for analysis. Descriptive statistics like frequency, percentage and mean were carried out for different variables. The association between two populations was cheeked using chi square test. Bi variable logistic regression analysis was used to know the crude association between each independent variables and outcome variable (stunting and wasting) and crude odds ratio was taken. Then variables which was associated with the dependent variable in bi-variable analysis with *p* value < 0.2 was included in the models of multivariable logistic regression analysis with backward likelihood ratio approach. Anthropometric data were converted in to indices and indicators using WHO Anthro software. *P*-value less than 0.05 in multivariable logistic regression analysis were used to conclude the presence of statistically significant association between different predictor variables with outcome variable (stunting and wasting). The strength of statistically association was measured by adjusted odds ratio at 95% confidence level.

### Ethical consideration

Ethical clearance was obtained from ethical Review Board of Bahir Dar University College of Medicine and Health Sciences (CMHS/ IRB 01–008) and zonal health department and selected kebele through formal letter. Before collecting the data, informed consent was obtained from all participants/parents/legal guardians. Each study participants were informed about the purpose of the study and participation was voluntary without payment for their participation. Each study participants also were informed that the right to withdraw at any time during the interview. All gathered information were protected from its confidentiality, anonymity was explaining clearly to participant. Except for the principal investigator, information is not exposed to a third person.

## Results

### Socio demographic and economic characteristics of study participants

The study included 648 mothers and their children aged 6–59 months (with a 95.0 percent response rate). The mean (+ SD) age of the mothers and children in households (HHs) with home gardening was 28.7(5.5) years and 30.8(12.6) months, respectively, and 29.7(5.8) years and 29.1 (10.3) months in households without home gardening. Almost sixty-eight percent (67.9%) of mothers from HHs with home gardening and eighty percent (79.9%) of mothers from HHs without home gardening had no formal education. Nearly, all mothers in both HHs (with home gardening = 99.4% and without home gardening = 98.4%) were married. The mean (± SD) family size in this study was 4.2(± 1.1) from HHs with home gardening and 4.3(± 0.9) from HHs without home gardening. Sixty nine percent (69.2%) from HHs with home garden and fifty four percent (54.5%) from HHs without home gardening were food secure (Table 1).

**Table1:** Distribution of households by selected socio-demographic and socio-economic characteristics in Zege, Northwest Ethiopia, 2020 (*n* = 616)

Characteristics	Households with home garden *N* = 308 (%)	Households without home garden *N* = 308(%)	Chi-square, *P*-value
Maternal age group (in year)			4.9,0.17
20–24	83(26.9)	69(22.4)	
25–29	95(30.8)	82(26.6)	
30–34	72(23.4)	91(29.5)	
> = 35	58(18.8)	66(21.4)	
Head of the household			28.9, < 0.0001
Male	242(78.6)	219(71.1)	
Female	41(13.3)	20(6.5)	
Both	25(8.1)	69(22.4)	
Decision making power of women			25.9, < 0.0001
Decide	244(79.2)	186(60.4)	
Not decide	64(20.8)	122(39.6)	
Religion			0.9, 0.3
Orthodox	290(94.2)	284(92.2)	
Muslim	18(5.8)	24(7.9)	
Marital status			1.3, 0.25
Married	306(99.4)	303(98.4)	
Single	2(0.6)	5(1.6)	
Resident			2.8, 0.09
Rural	262(85.1)	246(79.9)	
Urban	46(14.9)	62(20.1)	
Educational status of mother			2.5, 0.10
No formal education	209(67.9)	190(61.7)	
Primary and above	99(32.1)	118(38.3)	
Educational status of father			0.7, 0.38
No formal education	100(32.5)	109(35.4)	
Primary and above	199(64.6)	187(60.7)	
Mean age(SD) in month	30.86(12.596)	29.09(10.291)	16.8, 0.002
Sex of child			18, < 0.0001
Male	205(66.6)	153(49.7)	
Female	103(33.4)	155(50.3)	
Family size			0.52, 0.47
< 5	199(64.9)	191(62.0)	
> / = 5	108(35.1)	117(38.0)	
Occupation of mother			8.7, 0.032
Housewife	272(88.3)	246(79.9)	
Merchant	18(5.8)	27(8.8)	
Employed	10(3.2)	22(7.1)	
Student	8(2.6)	13(4.2)	
Occupation of father			15.2, < 0.0001
Farmer	230(77.2)	187(63.2)	
Merchant	23(7.7)	28(9.5)	
Employed	45(15.1)	81(27.4)	
Land size (in hectare)			0.1, 0.95
< 1	46(16.7)	39(16.5)	
1–2	163(59.1)	143(60.3)	
> 2	67(24.3)	55(23.2)	
Food security status of the household			13.9, < 0.0001
Secure	213(69.2)	168(54.5)	
Insecure	95(30.8)	140(45.5)	
Wealth index			4.5, 0.1
Poor	94(30.5)	112(36.5)	
Middle	100(32.5)	105(34.2)	
Rich	114(37)	90(29.3)	

### Health, Nutrition and WASH related characteristics

The majority of mothers from HHs who did not practice home gardening (96.1 percent) and those who did practice home gardening (88.0 percent) had an ANC visit. More than three-quarters (96.4%) of children in HHs without a home garden were fully immunized. Eighty-six percent (86.4%) of children from HHs with a home garden were breastfed exclusively. At 6 months, more than half of the children in both households (67.2 percent in HHs with a home garden and 66.2 percent in HHs without a home garden) had begun complementary feeding. The dietary diversity score among children was higher in HHs with a home garden (67.5%) than in HHs without a home garden (34.4 percent). The majority of (85.4%) HHs without home gardening and (66.6%) HHs with home gardening mothers practiced good hand wa(Table 2)

**Table2:** Distribution of households by selecting Health, Nutrition and WASH related characteristics in Zege, Northwest Ethiopia, 2020 (*n* = 616)

Characteristics	Households with home garden *N* = 308 (%)	Households without home garden *N* = 308(%)	Chi-square, *P*-value
**ANC visit**			13.8, < 0.0001
Yes	271(88.0)	296(96.1)	
No	37(12.0)	12(3.9)	
Place of delivery			12.5, < 0.0001
Home	43(14)	17(5.5)	
Health institution	264(86)	291(94.5)	
**Frequency of feeding**			3.7, 0.05
< 3	148(48.1)	124(40.3	
> / = 3	160(51.9)	184(59.7)	
**Fully immunized**			128, < 0.0001
Yes	179(58.1)	297(96.4}	
No	129(41.9)	11(3.6)	
**Take vitamin A**			4.4, 0.036
Yes	255(80.2)	269(87.3)	
No	53(17.2)	39(12.7)	
**Prelactal feeding**			9.1, 0.003
Yes	95(30.8)	131(42.5)	
No	213(69.2)	177(57.5)	
**Exclusive breast feeding**			2.4, 0.12
Yes	266(86.4)	252(81.8)	
No	42(13.6)	56(18.2)	
**Give colostrum**			1.6, 0.20
Yes	247(80.2)	259(84.1)	
No	61(19.8)	49(15.9)	
**Time to start complementary feeding**			0.06, 0.79
Timely start	207(67.2)	204(66.2)	
Not timely start	101(32.8)	104(33.8)	
**Presence of diarrhea within two weeks**			3.7, 0.052
Yes	26(8.4)	41(13.3)	
No	282(91.6)	267(86.7)	
**Shortness of breath within two weeks**			1.3, 0.23
Yes	21(6.5)	29(9.4)	
No	287(93.2)	279(90.6)	
Dietary diversity score			67.5, < 0.0001
> = 4	208(67.5)	106(34.4)	
< 4	100(32.5)	202(65.6)	
**Drinking water source**			33.3, < 0.0001
Protected	231(75.0)	284(92.2)	
Unprotected	77(25.0)	24(7.8)	
**Hand washing practice of mother**			29.3, < 0.0001
Good	205(66.6)	263(85.4)	
Poor	1o2(33.2)	45(14.6)	
**Presence of latrine**			4.1, 0.04
Yes	246(79.9)	265(86.0)	
No	62(20.1)	43(14.0)	

## Nutritional status of children from households with and without home gardening

**Fig. 1:**
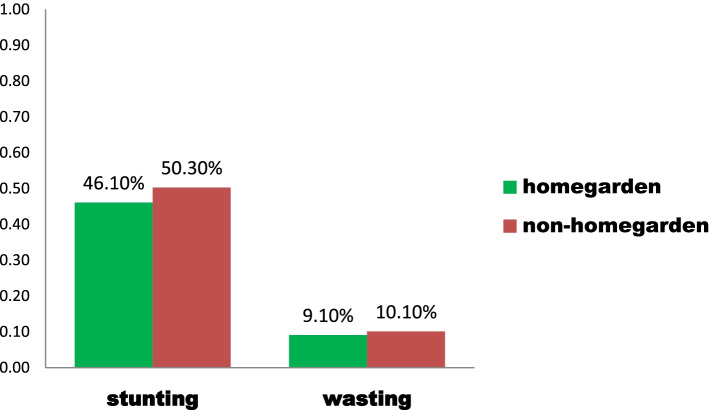
Prevalence of stunting and wasting from HHs with and without home gardening in Zege Northwest Ethiopia, 2020

About forty-six percent (46.1%) of children from HHs with home gardening and 50.30% from HHs without home gardening were stunted. Eighteen percent (18.2%) of children from home gardening and almost thirty percent (29.9%) from non-home gardening were underweight. Whereas nine percent (9.1%) of children from home gardening households and ten percent (10.1%) of children from non-home gardening households were wasted. (Fig. 1).

### Factors associated with stunting among children aged 6–59 months in Zege, Northwest Ethiopia

In the bivariate logistic regression model, age of mother, resident of respondent, age of child, sex of child, educational status of mother, drinking water source, presence of latrine, take vitamin A, immunization, Prelactal feeding, DDS and food security status of the household were associated with stunting at *P*-value < 0.2. After controlling for potential confounders, the final multivariable logistic regression analysis revealed that sex of child, feeding frequency, DDS and presence of diarrhea were factors significantly associated with child stunting.

The odds of male children to become stunted was 2.0 times higher than female children (AOR = 2. 0, 95% CI: 1.4–3.0). It was also observed that the odds of children who had less than 3 feeding frequency to become stunted was 1.6 times higher than children who had >  = 3 feeding frequency (AOR = 1.67, 95% CI: 1.1–2.4). Children who had diarrhea in the past two weeks prior to the study were 2.6 times more likely to be stunted than those children without diarrheal disease (AOR = 2.6, 95% CI: 1.4–4.6) and the odds of children who had low DDS to become stunted was 2.7 times higher than children who had high DDS (AOR = 2.7, 95% CI: 1.9–3.9) (Table 3).

**Table 3:** Factors associated with stunting among children aged 6–59 months in Zege, Northwest Ethiopia, 2020

Variables	Categories	Stunting		COR (95%CI)	AOR (95%CI)
		**Yes (%)**	**No (%)**		
**Residence**	Rural	257(50.6)	251(49.4)	1.7(1.1–2.6)*	1.2(0.7–1.9)
	Urban	40(37)	68(63)		1
**Educational status of mother**	No formal education	217(54.4)	182(45.6)	2.0(1.4–2.8)***	1.4(0.9–2.1)
	Primary and above	80(36.9)	137(63.1)		1
**Place of delivery**	Home	24(40)	36(60)	0.6(0.4–1.1)	0.5(0.2–1.0)
	Health institution	272(49)	283(51)		1
**Sex of child**	Male	208(58.1)	150(41.9)	2.6(1.8–3.6)***	2.0(1.4–3.0)***
	Female	89(34.5)	169(65.5)		1
**Feeding frequency**	< 3	178(51.7)	166(48.3)	1.3(1.002–1.8)	1.6(1.1–2.4)*
	> = 3	119(43.8)	153(56.2)		1
**Presence of latrine**	No	64(61)	41(39)	1.8(1.2–2.8)*	1.4(0.8–2.4)
	Yes	233(45.6)	278(54.4)		1
**Presence of diarrhea**	Yes	43(64.2)	24(35.8)	2.08(1.2–3.5) *	2.6(1.4–4.6) **
	No	254(46.3)	295(53.7)		1
**DDS**	< 4	190(62.9)	112(37.1)	3.2(2.3–4.5) ***	2.7(1.9–3.9) ***
	> / = 4	107(34.1)	207(65.9)		1
**Food security**	Insecure	130(55.3)	105(44.7)	1.5(1.4–2.2)*	1.2(0.8–1.7)
	Secure	167(43.8)	214(56.2)		1

^*^ = *P*-value < 0.05. ** = *P*-value < 0.01, *** = *P*-value < 0.001

### Factors associated with wasting among children aged 6–59 months in Zege, Northwest Ethiopia

Among the variables entered into bivariate logistic regression analysis, drinking water source, hand washing practice of mother, immunization status of child, sex of child, presence of latrine, time to start complimentary feeding, take vitamin A and exclusive breast feeding were associated with child wasting at *p*-value < 0.2. However, after controlling for potential confounders, the final multivariable logistic regression analysis revealed that sex of child, drinking water source, and immunization status were factors significantly associated with child wasting.

Therefore the odds of female children to become wasted was 2.4 times higher than male children (AOR=2.4, 95% CI: 1.3-4.6) and the odds of children who didn’t fully immunized to become wasted was 2.6 times higher than children who were fully immunized (AOR=2.6, 95% CI: 1.3-5.1). Moreover the odds of children from unprotected water source households to become wasted was 2 times higher than their counterparts (AOR=2.09, 95% CI 1.03-4.2) (table 4).

**Table 4:** Factors associated with wasting among children aged 6–59 in Zege, Northwest Ethiopia, 2020

Variables	Categories	Wasting		COR(95%CI)	AOR(95%CI)
		**Yes (%)**	**No (%)**		
**Sex of child**	Female	32(12.4)	226(87.6)	1.7(1.02–2.93) *	2.4(1.3–4.6) **
	Male	27(7.5)	331(92.5)	1	
**Hand washing practice**	Poor	21(14.3)	126(85.7)	0.52(0.3–0.9)*	0.7(0.4–1.4)
	Good	38(8.1)	430(91.9)	1	
**Immunization status**	Not immunized	23(16.4)	117(83.6)	2.4(1.3–5.10)**	2.6(1.3–5.1)**
	Immunized	36(7.6)	440(92.4)	1	
**Presence of latrine**	No	15(14.3)	90(85.7)	1.7(0.9–3)	0.94(0.4–1.9)
	Yes	44(8.6)	467(91.4)	1	
**Time to start complementary feeding**	Not timely	29(14.1)	176(85.9)	2(1.2–3.5)	0.7(0.38–1.3)
	Timely	30(7.3)	381(92.7)	1	
**Take vitamin A**	Yes	42(8.1)	477(91.9)	1	1.3(0.6–2.7)
	N0	17(17.5)	80(82.5)	2.4(1.3–4.4)**	1.3(0.6–2.7)
**EBF**	No	17(17.3)	81(82.7)	2.3(1.2–4.3) **	0.7(0.8–3.4)
	Yes	42(8.1)	476(91.9)	1	
**Drinking water source**	Unprotected	18(17.8)	83(82.2)	2.5(1.3–4.5)**	2.09(1.03–4.2)*
	Protected	41(8)	474(92)	1	

^*^ = *P*-value < 0.05, ** = *P*-vale, < 0.01, *** = *P*-value < 0.001

## Discussion

Agriculture, food security, and nutrition security are all intertwined issues in the human development [32]. Children's nutritional status is the result of complex interactions between food consumption, agricultural products, and the overall health status and health care practices [33]. Given this, increased agricultural productivity can contribute to improved livelihoods, including increased food consumption. The purpose of this study was to compare the nutritional status of children aged 6–59 months in Zege, North West Ethiopia, between households that practiced home gardening and those that did not.

In this study, The overall prevalence of stunting is 48% (95%, CI: 44.3–52.2). The finding are consistent with those of numerous studies conducted in various parts of the country [8, 9, 22, 23, 27]. However, The prevalence is higher than in the Amhara region and the country value [11] and study done on Nepal and Nigeria [33, 34]. Stunting indicated a lack of adequate food over a long period of time, which was exacerbated by infections. Despite minor improvements in the 2019 EDHS report, the current prevalence of stunting is remains a public health issues in the study area. This disparity in prevalence difference could be explained by difference in the socioeconomic status, agricultural productivity, food insecurity at household level, and intra household resource allocation. The diversity in agricultural production and livestock ownership enhance individual dietary diversity outcomes in young children aged 6–23 months [35, 36]. Another reason for this could be a difference in cultural and child feeding habits, study setting, and study periods [37, 38].

The prevalence of wasting in this study was comparable to the regional and the national values [11] and other parts of the country such as, rural Ethiopia,South Ethiopia, North west Ethiopia,East Gojam zone and Northeast Thailand [8, 22, 27, 39, 40] but the result was lower than other studies like Pastoral communities of Afar, Meta-Analysis conducted in Ethiopia,Nepal, Nigeria and Maharashtra [9, 23, 33, 34, 41]. Wasting is an indicator of acute malnutrition which can occur as result recent infection or as result weight loss due to seasonal variation of food supply. This difference could be explained by difference in the sample- size, socioeconomic, socio –cultural, feeding habits, study area and period of the study population.

According to the current study, there is no significance difference in child nutritional status between households with and without home gardening practice [15, 42]. However, Studies conducted in Southern Ethiopia and Zimbabwe revealed a signifcant relationship between home gardening and child nutrition [27, 43]. This could be due to participants' consumption of monotonous type habit. Production alone does not guarantee for good nutritional status unless it is supplemented with dieversified poducion and consumption of these products [36].

In the current study, male children were more likely to be stunted than female children. Male children had a 3.1 times greater chance of becoming stunted than female children but female children had a 2.4 times greater chance of being wasted. This could be explained partially by the fact that boys are more vulnerable to health inequalities than their female counterparts in the same age groups and there may be biological differences that boys are more susceptible to infectious diseases and show higher biological fragility in the first year of life [44, 45]. It could also be explained by unmeasured factors such as parental gender preference or sex difference in feeding practice[9]. Many studies in Ethiopia, Afar regional state [9], Meta-analysis in Ethiopia [23], South Ethiopia [27] East Hararghe Zone and South Wollo Zone [46] and EDHS 20,119 report [11] revealed the same finding.

In the current study, children’s immunization status was found to have a significant association with increased risk of high prevalence of stunting and wasting from gardening households. Non-immunized children were 1.9 and 6.2 times more likely to become stunted and wasted than immunized children respectively. This could be explained by the fact that non immunized children are at risk of many vaccine preventable diseases such as diarrhea and respiratory infections, which lead to nutrients depletion in the body. This finding was consistent with other studies conducted in pastoral communities of Afar [9], EDHS 2016 report [47] and Nepal [33].

Prelactal feeding was found to be another factor associated with stunting in the current study. The risk of stunting was 1.9 times higher in children who received prelactal feeding at birth than in children who did not receive prelactal feeding at birth. This could be because it has a negative impact on breastfeeding, and when children are not breastfed properly, they are at higher risk of developing malnutrition [9].

In this study, dietary diversity was found to be associated with child stunting in both households with and without home gardening practice. Children with low DDS were more likely to be stunted than children with high DDS. This could be explained by saying that children who ate monotonous or limited variety of foods were at risk of micro nutrient deficiency which could lead to chronic malnutrition. This finding was supported with study done in Meta-Analysis conducted in Ethiopia [23],Study conducted at south Ethiopia [27], Study conducted on rural Myanmar [48] and South Africa [15].

The presence of diarrheal disease was found to be significantly associated with an increase prevalence of stunting in the current study. Children who had diarrheal disease in the past two weeks prior to the study were 3.2 times more likely to be stunted than those children without diarrheal disease. According to UNICEF, this could be because diarrhea causes a decrease in appetite, poor digestion, and malabsorption, all of which lead to malnutrition [49].This result was consistent with other findings conducted in south Ethiopia [27]**,** Meta-Analysis conducted in Ethiopia [23], EDHS 2016 report [47] and South Africa [15].

In the current study, a child's feeding frequency was also significantly associated with an increase in the prevalence of stunting in non-gardening households. Children who had fewer than three feedings per day were 1.7 times more likely to be stunted than children who had more than three feedings per day. This could be because low feeding frequency leads to insufficient nutrient intake, which leads to malnutrition. This result was consistent with the findings of a study conducted in Northwest Ethiopia [50].

Drinking water source was found to be a significant association with increased risk of wasting in this study. Children in households with unprotected water source were 2.5 times more likely to be wasting than children in households with protected water source. This could be due to the fact that water from a contaminated source may serves as a source for different parasitic and gastrointestinal infection, which causes children to be malnourished (wasting) [49]. This finding was supported by research conducted in the East Go jam zone [8], the EDHS 2016 report [47] and rural Ethiopia [22].

## Conclusions and recommendations

This study found that there was no statistically significance difference in nutritional status of under-five children from households with and without home gardening. The significant predictors of nutritional status (stunting and wasting) were sex of child dietary diversity score, immunization status, drinking water source and, feeding frequency, and presence of diarrhea.

Prevention and control of childhood illness such as diarrheal through improving environmental health conditions such as provision of safe and adequate water, prevention of contamination of food and water and health care services. Incite heath extension workers to address vaccine preventable disease integrated with the access of nutrition education programs to improve feeding frequency and dietary diversity of children are recommended activities to reduce malnutrition among under five children.

To researchers: Farther nutritional status studies for under –five children on different seasons and repeated recall days for identify dietary diversity of children, in addition to that studies on maternal awareness and attitude on proper use of home gardening products to child nutrition.

### Limitations of the study

This study should be interpreted in the context of the following limitations. There may be a recall bias during data collection time that might be over and under estimate the finding and the Dietary intake was assessed using a single day recall which may not show permanent habit of the households. The study may not free from social desirability bias in responding to questions on house hold food security.

## Data Availability

All the required data has been included in the manuscript.

## Abbreviations

AOR: Adjusted Odds Ratio
CI: Confidence Interval
COR: Crude Odds Ratio
DDS: Dietary Diversity Score
EDHS: Ethiopia Demographic Health Survey
FANTA: Food and Nutrition Technical Assistance
FAO: Food Agricultural Organization
FNP: Food and Nutrition Policy
G.C: Gregorian Calendar
GDP: Gross Domestic Product
HEW: Health Extension worker
HHS: Households
HAZ: Height for Age Z score
NNP: National Nutrition Program
OR: Odds Ratio
SD: Standard Deviation
SDG: Sustainable Development Goal
SPSS: Statistical Package for Social Sciences
UNICEF: United Nations International Children’s Emergency Fund
WAZ: Weight for Age Z score
WHO: World Health Organization
WHZ: Weight for Height Z score
